# The Influence of Isoflurane Anaesthesia on the Rat Grimace Scale

**DOI:** 10.1371/journal.pone.0166652

**Published:** 2016-11-17

**Authors:** Amy L. Miller, Huw D. R. Golledge, Matthew C. Leach

**Affiliations:** 1 School of Agriculture, Food and Rural Development, Agriculture Building, Newcastle University, Newcastle upon Tyne, NE1 7RU, United Kingdom; 2 Institute of Neuroscience, The Medical School, Newcastle University, Newcastle upon Tyne, NE2 4HH, United Kingdom; Central South University, CHINA

## Abstract

Over 234,000 rats were used in regulated procedures in the UK in 2014, many of which may have resulted in some degree of pain. When using animals in research, there is an ethical and legal responsibility to alleviate or at least reduce pain to an absolute minimum. To do this, we must be able to effectively assess pain in an accurate and timely manner. The Rat Grimace Scale (RGS) is a pain assessment tool, which is suggested to be both accurate and rapid in pain assessment. Many procedures involve the use of general anaesthesia. To date, the effects of anaesthesia on the RGS have not been assessed, limiting its potential utility for assessing pain following anaesthesia. Forty-eight Lister hooded rats were used in this study (24 in part A and 24 in a separate part B). Rats were randomly assigned to one of two treatment groups in part A; short duration isoflurane exposure, short duration control exposure (air) and one of two treatment groups in part B; surgical duration isoflurane exposure or surgical duration control exposure (oxygen). Rats were placed into an anaesthetic induction chamber and isoflurane, or control gas piped into the chamber for either 4 (short duration exposure) or 12 minutes (surgical duration exposure). Following recovery, photographs of the rats’ faces were taken and then scored blindly using the RGS. Short duration isoflurane anaesthesia had no effect on RGS scores. However, when rats are anaesthetised for a longer duration, akin to a simple routine surgical procedure, the RGS score increases significantly and this increase remains on repeated exposure to this duration of anaesthesia over a 4-day period. This should be accounted for when using the RGS to assess pain in rats in the immediate time period following procedures involving the use of isoflurane anaesthesia.

## Introduction

Pain assessment in *in vivo* research is essential to ensure that both welfare and ethical and legal standards are met. In 2014, over 234,000 rats were used in scientific procedures in the UK [1]. Many of these regulated procedures are likely to be associated with some degree of pain and distress and therefore must be accompanied by the use of anaesthesia or analgesia unless there is specific justifiable scientific reasoning to withhold them. In order to effectively assess the pain associated with such procedures pain assessment tools need to be effective, reliable and valid. A recent addition to the pain assessment toolkit for rats is the Rat Grimace Scale (RGS) developed by Sotocinal et al. [2]. The RGS consists of 4 facial action units (FAUs); orbital tightening, nose & cheek flattening, ear changes and whisker changes. Each of these AUs is scored on a 3-point scale (0 = not present, 1 = moderately present & 2 = obviously present). The sum of the scores recorded for each FAU can then be used in analysis. The rat grimace scale, along with the other recently developed grimace scales for other species have been proposed as a reliable and effective means of assessing pain [2]. However, for these techniques to be considered valid, particularly for application in clinical scenarios, then the influence of other non-painful procedures that are integral to the research being carried out (e.g. handling, anaesthesia or analgesia) needs to be understood and taken into account in any assessment of pain. Recently, Miller et al [3] demonstrated that isoflurane anaesthesia alone (i.e. in a non-painful state) increased Mouse Grimace Scale (MGS) score in male DBA/2 mice.

Isoflurane is a very widely used inhalation anaesthetic in *in vivo* research, as it produces rapid induction and recovery from anaesthesia and undergoes less biotransformation than other agents making it a suitable choice in a range of *in vivo* studies [4]. This routinely includes very short procedures e.g. implanting transponders (e.g. [5]) or certain injections (e.g. [6]) through to longer duration procedures such as surgery (e.g. [7,8]). Volatile anaesthetics have a high fat/blood coefficient, however absorption into the fat is slow in comparison to other tissues. During very short procedures, limited anaesthetic will be absorbed into the fat, however, during longer procedures, increased amounts of anaesthetic would be absorbed into the fat leading to an increase in recovery time because anaesthetic is released from fat following discontinuation of anaesthetic inhalation [9]. This increased recovery time has the potential to lead to some residual effects when monitoring of pain early in the post procedural period due to the increased sedation reducing the exhibition of pain indices, including specific pain behaviours, activity [10] and potentially facial expressions.

In addition, due to technology advances, repeated procedures involving isoflurane anaesthesia may be increasingly carried out, for example in tumour models in which *in vivo* imaging of the rodent at certain time point allows for accurate quantification of tumour burden, without the need to euthanize the animal [6] but where the animal must be immobilised to allow image capture. Longitudinal monitoring of individual animals has large, beneficial impacts in terms of 3Rs. The ability to study one animal at multiple time points reduces the overall number of animals required and with each animal acting as its own control, decreased variation within groups (Reduction). However, re-exposure to isoflurane is known to be more aversive than initial exposure to isoflurane in rats [11] and therefore may act as a confounding factor on the assessment of pain if behaviour is altered.

Currently, we do not know if the duration of isoflurane anaesthesia and/or repeated exposure to isoflurane alone has an influence of the RGS and if so the magnitude of this effect. Here we aim to study the effect of both short-duration and surgical-duration anaesthesia at a depth which would typically be used for scientific procedures on baseline (non-painful) RGS scores. In addition, we aim to determine if repeated exposure to isoflurane anaesthesia resulted in changes in RGS score over time.

## Materials and Methods

All procedures were conducted in accordance with the Animals (Scientific Procedures) Act 1986, European Directive EU 2010/63 and with the approval of the Animal Welfare and Ethics Review Board at Newcastle University.

### Animals

Forty-eight male hooded Lister rats (Charles River, Kent, UK) aged 8 weeks were used. Rats were housed in groups of 4 in 12 cages. All cages contained sawdust bedding and nesting material (DBM Ltd, Edinburgh, UK). Cardboard tubes and chew blocks (Datesand, UK) were provided as enrichment. Food (RM3, SDS UK) and tap water was provided *ad libitum*. The animal room was maintained at 21°C ± 1°C, 48% humidity and on a 12/12 hour light dark cycle (lights on at 07:00). A seven-day acclimatisation period was given prior to the start of the study. Twenty four rats were randomly allocated, using a random number generator, to one of two treatment groups (n = 12/group) in Part A of the study; group 1—Short duration Isoflurane exposure (4 minutes), group 2—Short duration air exposure (4 minutes). Twenty four rats were randomly allocated, using a random number generator, to one of two treatment groups (n = 12/group) in Part B of the study; group 3—Surgical duration isoflourane exposure (12 minutes) or group 4—Surgical duration oxygen exposure (12 minutes). The animals were free from any common pathogens in accordance with the FELASA health monitoring recommendations.

### Part A: Short duration exposure

Rats in group 1 (short duration isoflurane) and group 2 (short duration air) were individually transferred to a procedure room and placed into an anaesthetic induction chamber (30cm x 20cm x 17cm). For group 1, anaesthesia was induced with 5% isoflurane in oxygen (2.4l/min) for 2min. Anaesthesia was maintained with 2% isoflurane in oxygen (2.4l/min) for a further 2 minutes. Animals in group 2 underwent an identical procedure but rather than isoflurane and oxygen being delivered to the induction chamber, medical air was delivered at an equivalent flow rate to that of the anaesthetic and oxygen (group 1). Following recovery (approximately 15 minutes) from exposure to either isoflurane or air, rats were transferred to a quiet room and placed in a photography box (30 x 20 x 20cm) for a period of 5 minutes and photographed using a high definition camera (Casio EX-ZR100, Casio Computer Co., Ltd., Japan) by a treatment-blinded observer. The photography box consisted of two matte black walls and two clear perspex walls (27cm x 19cm x 17cm). Rats were photographed on every occasion they directly faced the camera, apart from when grooming in accordance with the method set out by Sotocinal et al [2]. After 5 minutes, the rats were returned to their home cages. The box was then thoroughly cleaned and dried before the next rat was placed inside in order to remove any odour. This process of exposure to isoflurane or air followed by photographing of the face was repeated daily for 4 consecutive days at the same time each day.

### Part B: Surgical duration exposure

Rats in group 3 (surgical duration isoflurane) and group 4 (surgical duration oxygen) were individually transferred to a procedure room and placed into the anaesthetic induction chamber. Anaesthesia was induced with 5% isoflurane in oxygen (4l/min). When the tail pinch reflex was absent, anaesthesia was maintained with 2% isoflurane in oxygen (2l/min) for 12 minutes. Animals in group 4 underwent an identical procedure without isoflurane, with oxygen being delivered to the induction chamber at an equivalent flow rate to that used for group 3. Following recovery from anaesthesia (15 minutes) the process of photographing the rats was carried out in the same manner as described above. This process of exposure to isoflurane or oxygen followed by photographing of the face was repeated daily, for 4 consecutive days, at the same time each day.

### Data collection

The collected images were cropped, leaving only the face of the rat in view to prevent bias due to body posture [12]. Using a random number generator, three images per rat, per time point were selected. Using the random number generator again, the selected images were re-ordered and inserted into a custom designed excel file for scoring. Trained observers who were blinded to the experimental details, design and purpose scored each photograph for the four facial action units (FAUs) comprising the RGS as described by Sotocinal et al [2]. For each image, the 4 individual FAUs; orbit tightening, nose / cheek flattening, ear changes and whisker change were scored using a 3-point scale (0 = not present, 1 = moderately present, 2 = obviously present). The RGS manual was provided to the scorers for reference, but the title of the manual was edited to ‘rat facial action coding manual’ to limit bias of scores from the title. Scores for each FAU, for each individual image were then combined by simply totalling the individual scores to produce a composite grimace score for each image.

### Statistical analysis

Data were analysed non-parametrically using SPSS software (version 21, IBM). A Mann-Whitney U test was used to compare the two short duration treatment groups (groups 1 and 2) with each other and the two long duration treatment groups (groups 3 and 4) with each other on each individual test day (Days 1–4). A Friedman’s test was used to compare the scores across time for each treatment group (groups 1–4). Significant differences between time points were compared using a Wilcoxon test with an adjusted Bonferroni correction for multiple comparisons being applied where appropriate. Differences were considered to be statistically significant if P<0.05.

Following conclusion of this study, those rats in the control groups went on to be used in an unrelated research project. Those rats that had had multiple exposures to isoflurane anaesthesia were euthanized using overdose of CO_2_.

## Results

### Short duration exposure

**Fig 1:**
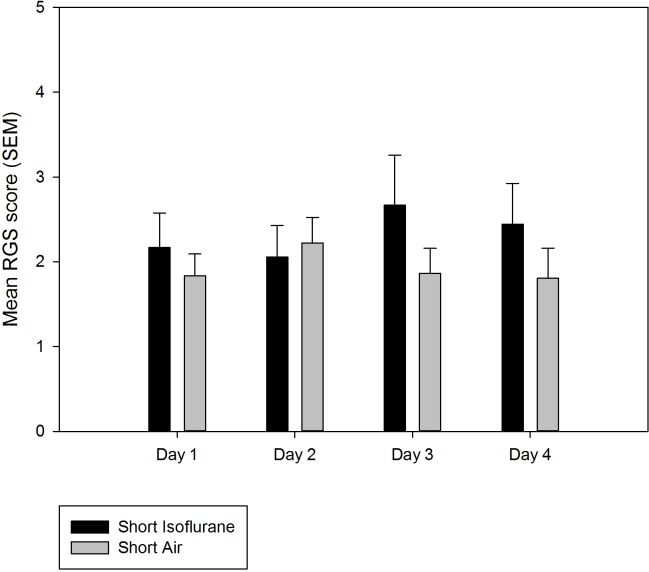
Mean rat grimace scale score (± SEM) for animals in the short duration isoflurane and air groups on 4 consecutive days. Maximum score8.

There was no significant difference in RGS score between groups 1 (short duration isoflurane) and 2 (short duration air) on any of the trial days. There was also no significant difference in RGS score between the four trial days within either the short duration isoflurane group (group 1) or short duration air group (group 2) (Fig 1).

### Surgical duration exposure

**Fig 2:**
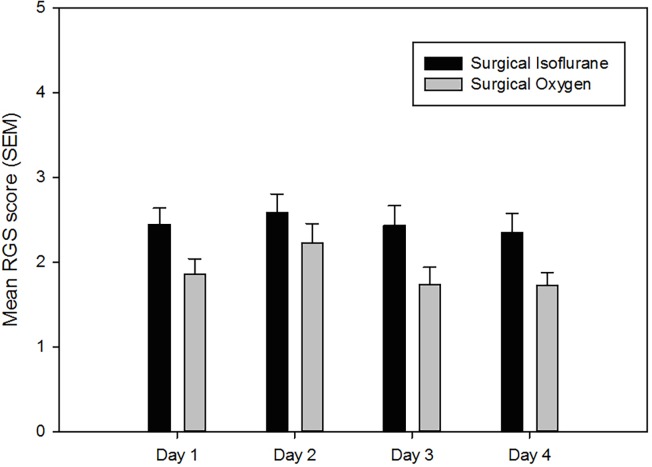
Mean rat grimace scale score (± SEM) for animals in the surgical duration isoflurane and oxygen groups on 4 consecutive days. Maximum score 8.

There was a significant difference in RGS score between groups 3 (surgical duration isoflurane) and 4 (surgical duration oxygen) on some of the trial days. On days 1, 3 and 4, group 3 (surgical duration isoflurane) had a significantly greater RGS score than group 4 (surgical duration oxygen) (Fig 2). On day 2, there was no significant difference between the two groups. There was no significant difference in RGS score between the four trial days within either surgical duration isoflurane group (group 3) or the surgical duration oxygen group (group 4). The mean scores for each individual action unit are shown in Table 1.

**Table 1:** The mean scores for each individual action unit, on each day for each surgical duration treatment group.

Treatment day / Group	Orbital tightening	Nose & ear flattening	Ear change	Whisker change
Day 1 –Oxygen	0.333	0.611	0.556	0.361
Day 1 –Isoflurane	0.319	0.806	0.806	0.514
Day 2 –Oxygen	0.333	0.722	0.667	0.5
Day 2 –Isoflurane	0.389	0.875	0.667	0.653
Day 3 –Oxygen	0.333	0.514	0.514	0.375
Day 3 –Isoflurane	0.431	0.722	0.681	0.575
Day 4 –Oxygen	0.278	0.556	0.431	0.389
Day 4 –Isoflurane	0.236	0.792	0.875	0.528

## Discussion

The Rat Grimace Scale (RGS) has shown promise as a novel means of assessing pain in rats [2]. Sotocinal et al [2] demonstrated the RGS to be reliable and accurate in the quantification of the time course of pain in both multiple nociceptive models and changes in response to surgery (laparotomy) with post-surgical scores reducing in a dose dependent manner with morphine analgesia administration. In this study, we have investigated the influence of two durations of isoflurane anaesthesia in non-painful rats to establish whether brief and surgical duration isoflurane anaesthesia influences exhibition of the RGS in a common laboratory rat strain. Understanding the degree of effect, if any, of this routine but non-painful procedure, that is often integral to the research carried out, is critical to further validation of the RGS. This is particularly important if grimace scales are to be implemented clinically where baseline grimace scores for individuals (prior to handling, anaesthesia, surgery etc.) are not available as they would be in a research setting (e.g. [12]). Additionally we cannot assume that baseline RGS will be zero. A recent study has demonstrated variation in baseline grimace scores in mice between both strains and sexes [13]. Therefore establishing what the baseline score for a given strain of rat is, and ensuring that it is consistent between individuals, would be imperative for this tool to used in a clinical setting.

RGS scores following 4 minutes of isoflurane anaesthesia (short duration) were equivalent to scores in control rats and did not change significantly following repeated (daily for 4 days) short duration isoflurane anaesthesia. Therefore, the RGS would appear in this respect to be a valid means of assessing pain in rats where anaesthesia was for less than 4 minutes. In contrast however, rats that had been exposed to 12 minutes isoflurane anaesthesia at a duration and depth equivalent to that used for a routine surgical procedure (e.g. laparotomy), demonstrated significantly higher RGS score in comparison to controls and this score remained elevated compared to the controls following repeated (daily for 4 days) surgical duration isoflurane anaesthesia. This level of increase would need to be quantified and deemed consistent across multiple cohorts, within a given strain of rats before this tool could be considered for clinical use or when comparing change in RGS score between various procedures. Additionally, the duration of time following anaesthesia when increased RGS scores are observed should also be quantified in order for this assessment tool to be used appropriately. Furthermore in real-world situations the depth of anaesthesia used and the duration of anaesthesia for surgery is likely to vary between individuals, introducing an additional potential source of variability in grimace scale ratings. Therefore, additional study is required to prior to the RGS being implemented as a clinical pain assessment tool following isoflurane anaesthesia.

Following an initial exposure to isoflurane, rats show an increase in averseness during subsequent exposures [11]. Here, we studied if this known increase in averseness had any impact on RGS score following repeat exposure to isoflurane. If so, this could impact on its usefulness in pain assessment.

Our results demonstrate that the RGS scores were not influenced by repeat exposure (4 separate occasions) to isoflurane, which simulates longitudinal monitoring via a scanning technique. This is an important finding with respect to the use of the RGS in pain assessment for either clinical use or as a research tool when longitudinal monitoring is required.

## Conclusion

Short duration isoflurane anaesthesia (4 minutes) does not have any effect on RGS scores in male Lister hooded rats. However, when these rats are anaesthetised for a longer duration (i.e. 12 minutes), akin to a simple routine surgical procedure (i.e. laparotomy) or scanning procedure, the RGS score increases significantly and this increase remains on repeated exposure to this duration of isoflurane anaesthesia over a period of 4 days. This must be taken into account when using the RGS to assess pain in rats in the immediate time period following procedures involving the use of isoflurane anaesthesia.

## Supporting Information

S1 File Data used in analysis and used to generate figures. (XLSX)
